# Lipid droplets in the endothelium: The missing link between metabolic syndrome and cardiovascular disease?

**DOI:** 10.1172/JCI176347

**Published:** 2024-02-15

**Authors:** Iris Z. Jaffe, S. Ananth Karumanchi

**Affiliations:** 1 Molecular Cardiology Research Institute, Tufts Medical Center, Boston, Massachusetts, USA.; 2 Department of Medicine, Cedars-Sinai Medical Center, Los Angeles, California, USA.

## Abstract

The physiology of lipid droplets (LDs) has been most extensively characterized in adipocytes, but LDs also accumulate in endothelial cells lining blood vessels in response to changing levels of triglycerides. In recent issues of the *JCI*, two independent papers highlight a direct role of endothelial LDs in the genesis of hypertension and atherosclerosis in rodent models. Kim et al. demonstrated that accumulation of LDs in the endothelium leads to hypertension, impairs endothelial function, and accelerates atherosclerosis. Boutagy, Gamez-Mendez, et al. knocked out *Atgl* in the endothelium and confirmed triglyceride accumulation in endothelial cells that was associated with reduced NO synthesis and impaired endothelial-dependent vasodilation. These data suggest that enhancing triglyceride breakdown in the endothelium could provide a treatment target for patients with metabolic syndrome.

## The pathophysiologic impact of excessive fatty acids

Lipid droplets (LDs) are intracellular fat reservoirs that store neutral lipids for future use as an important energy source in metabolically active tissues (1, 2). The physiology of LDs has been most extensively characterized in adipocytes and, in general, is thought to provide fuel for metabolic processes and membrane biosynthesis during periods of nutrient deprivation. Incorporation and release of fatty acids from LDs occurs via established enzymatic pathways, with adipose triglyceride lipase (ATGL) being the rate-limiting enzyme for LD hydrolysis. Further, by sequestering fatty acids as triacylglycerol (also known as triglycerides [TGs]), LDs protect against the deleterious effects of free fatty acids. Impairment of formation of LDs plays a critical role in diseases such as lipodystrophy in which the reduced ability to store lipids leads to insulin resistance (3). Excessive LD formation occurs in diffuse tissues in rare conditions involving mutations resulting from loss of function of lipolysis enzymes including ATGL (2, 4). However, beyond rare mutations, the pathophysiologic impact of excessive fatty acids is commonly associated with highly prevalent problems of obesity and metabolic syndrome. Metabolic syndrome is a constellation of findings including high circulating TGs that is associated with hypertension and increased cardiovascular disease risk (5). It has recently been shown that LDs accumulate in endothelial cells lining the blood vessels in response to changing levels of TGs in vivo; however, their physiological role was not known (6). Additionally, whether endothelial LD formation is a consequence of metabolic syndrome and its direct contribution to cardiovascular disease have not been previously explored.

## Endothelial LDs contribute to hypertension and atherosclerosis

In this issue of the *JCI*, two papers highlight a direct role of endothelial LDs in the genesis of hypertension and atherosclerosis in rodent models. Kim et al. demonstrate that accumulation of LDs in the endothelium leads to hypertension, impairs endothelial function, and accelerates atherosclerosis (7). To induce endothelial LDs in mice, the authors fed mice a high-fat diet or tested mice that lacked *Atgl* in the endothelium, which specifically induced LD formation in endothelial cells. Interestingly, in both these models of LD accumulation in the endothelium, the animals developed an increase in blood pressure without other features of metabolic syndrome, such as hyperinsulinemia. The authors found excess LDs in the endothelium was associated with impairment of nitric oxide (NO) synthesis, an important regulator of blood pressure. The mechanism involved reduced stability of the mRNA for the NO-producing enzyme endothelial nitric oxide synthase (eNOS). The resulting reduction in eNOS protein and NO production was associated with impaired endothelium-dependent vasodilatory response. Further, the authors demonstrated that excess LDs in the endothelium activated proinflammatory pathways in vitro by activating NF-κB. They also showed that enhanced atherogenesis occurred in vivo. When endothelial LDs were normalized, hypertension and inflammation resolved, supporting the concept of causation rather than correlation.

**Figure 1:**
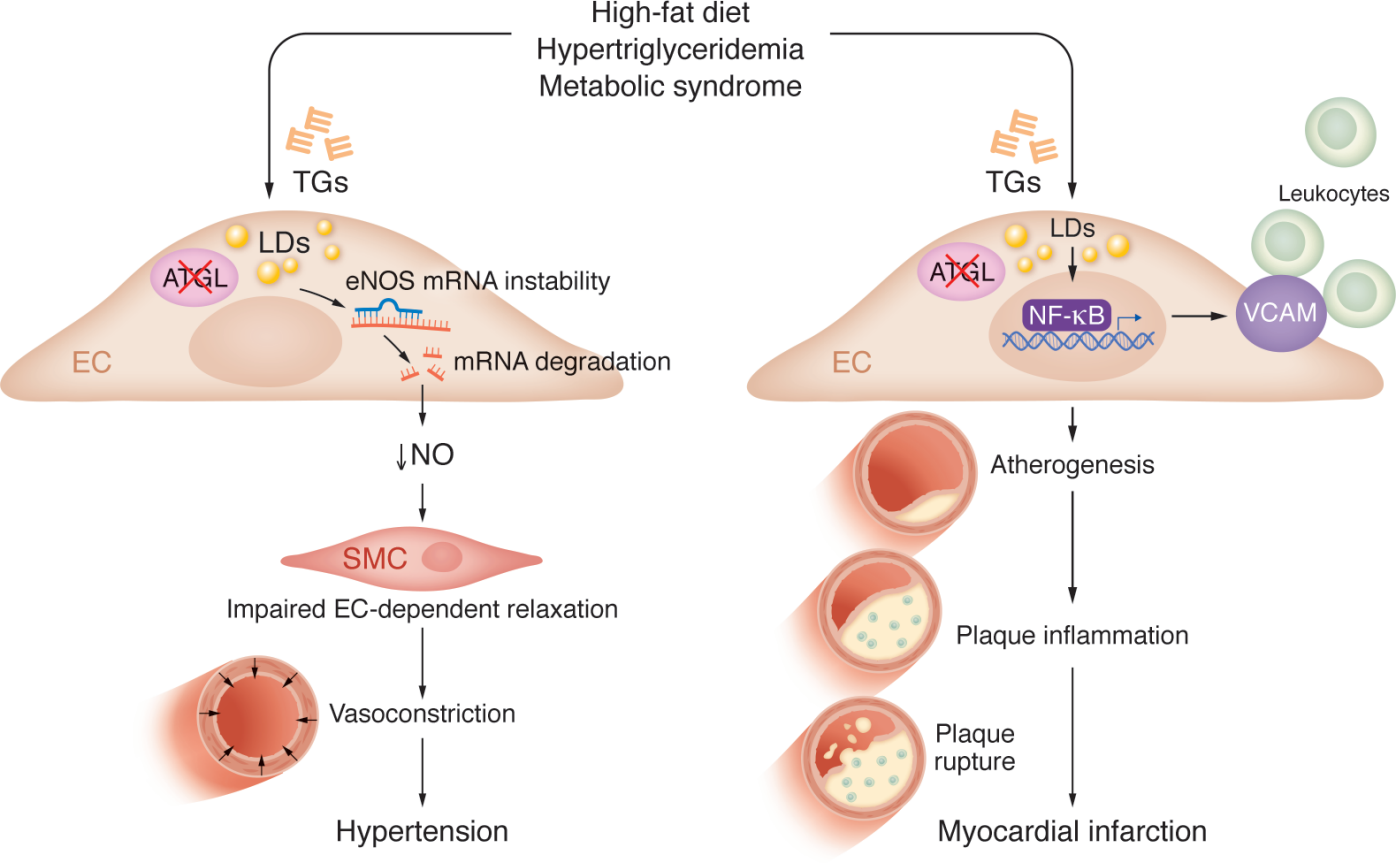
Accumulation of LDs in the endothelium promotes atherosclerosis and hypertension. A high-fat diet or loss of ATGL in endothelial cells (ECs) results in the formation of TG-rich LDs in the endothelium. These LDs may contribute to cardiovascular disease via the suppression of NO production or through vascular cell adhesion molecule–mediated (VCAM-mediated) vascular inflammation. Decreased NO impairs EC-mediated relaxation, resulting in enhanced vasoconstriction, which contributes to hypertension. In parallel, endothelial LDs activate NF-κB, which induces expression of the adhesion molecule VCAM. Notably, VCAM contributes to leukocyte-EC adhesion, atherogenesis, and plaque inflammation, all of which can lead to plaque rupture and myocardial infarction.

In an independent report, Boutagy, Gamez-Mendez, et al. also deleted *Atgl* in the endothelium and confirmed TG accumulation in endothelial cells that was associated with reduced NO synthesis and impaired endothelial-dependent vasodilation (8). The authors demonstrated that loss of *Atgl* in endothelial cells led to profound inflammation with increased surface expression of leukocyte adhesion molecules on endothelial cells. This response was associated with enhanced atheroma lesion formation with more inflamed plaques in a mouse model of atherosclerosis. Plaque inflammation is known to be associated with increased risk of rupture, the predominant cause of myocardial infarction in humans. Together, these two manuscripts demonstrate a pathological link between neutral lipid and LD accumulation in endothelial cells with vascular pathologies (Figure 1).

## Clinical implications and conclusions

What is the importance of these findings? In addition to confirming the known link between high-fat diet and TG-rich endothelial LDs (2, 6), the data provided in Kim et al. (7) and Boutagy, Gamez-Mendez, et al. (8) reveal a mechanistic link between high-circulating, TG-rich lipoproteins and common vascular diseases that is mediated by induction of LDs in the endothelium. Specifically, the observation that endothelial LDs suppress NO synthesis and induce a proinflammatory state provides a mechanistic link between endothelial LDs and both hypertension and atherosclerosis. Further, these papers highlight a pathogenic role of TG-rich LD in the context of ATGL deletion, in contrast to the more traditionally accepted view where TG synthesis is thought to buffer against lipid-induced ER stress. A direct role of TG levels in cardiovascular disease has been controversial, as mechanistic studies have been lacking. While high circulating LDL cholesterol is causally linked with atherosclerosis, there is still residual risk for cardiovascular disease in patients taking statins, which effectively lower LDL cholesterol. Recent data in humans with well-treated LDL but high TGs have shown that TG lowering itself with icosapent ethyl reduced acute cardiovascular ischemic events, suggesting that additional cardiovascular protection may occur from lowering TGs (9). Since acute cardiovascular events, such as unstable angina, ischemic stroke, and acute myocardial infarction, are caused by rupture of inflamed atherosclerotic plaques, both these manuscripts now provide mechanisms for how a high-fat diet and high TGs can contribute directly to hypertension and vascular inflammation via LD toxicity in the endothelium. However, there are still several unanswered questions. Interestingly, humans with *ATGL* mutations have been described, and these patients present with hepatomegaly, myopathy, and cardiomyopathy, but not specifically with hypertension and/or excess atherosclerosis (4). Whether this outcome is related to maintenance of some residual functional activity for the enzyme in the endothelium or some counter effect of ATGL deficiency in other tissues is not known. Interestingly, global knockout of *Atgl* in mice phenocopies aspects of the human syndrome, including cardiac steatosis and severe heart failure; moreover, global *Atg–*deficient mice suffered from pronounced vascular endothelial dysfunction that was rescued by PPARα agonists, which restored NO synthase enzyme activity (10).

Although both manuscripts showed that LD accumulation in the endothelium associated with decreased eNOS expression, NO production, and impaired endothelium-dependent relaxation, the direct mechanisms remain to be determined (7, 8). In addition, Kim et al. (7) further shows that these characteristics are associated with increased systemic blood pressure. However, both groups measured endothelial vasodilation in conduit vessels (aorta, carotid), which do not contribute to regulation of blood flow or blood pressure control. Examination of endothelial function in resistance vessels, which contribute to peripheral vascular resistance and hence blood pressure, would provide stronger evidence that impaired endothelial NO production mediates the blood pressure response in these models. A more substantial limitation is that both manuscripts studied the impact of LDs in endothelial cells using exclusively male animals. As such, whether these mechanisms are relevant in females cannot be inferred from either study. Obesity disproportionately affects women (11) and, when associated with metabolic syndrome, has a greater negative impact on cardiovascular outcomes in women (12). Hence, understanding molecular mechanisms linking dyslipidemia to cardiovascular disease in females is particularly important.

In sum, TG-rich LDs that form in the endothelium as a consequence of high-fat diet or due to loss of ATGL may contribute directly to cardiovascular disease by inducing vascular inflammation and promoting atherosclerosis and by suppressing NO formation to contribute to hypertension (Figure 1). The metabolic syndrome that consists of lipid changes, including raised TGs, low HDL cholesterol, and hypertension and insulin resistance, is very common in the adult population throughout the world (5). The treatment of metabolic syndrome currently requires separate agents for treating dyslipidemia and hypertension. These data suggest that enhancing TG breakdown in the endothelium could be a therapeutic target for patients with metabolic syndrome that may provide multiple benefits, and hence, warrants further exploration.

## Version 1 02/15/2024

Electronic publication
